# A Reinvestigation of a Superhard Tetragonal *sp*^3^ Carbon Allotrope

**DOI:** 10.3390/ma9060484

**Published:** 2016-06-17

**Authors:** Mengjiang Xing, Binhua Li, Zhengtao Yu, Qi Chen

**Affiliations:** Faculty of Information Engineering and Automation, Kunming University of Science and Technology, Kunming 650051, China; ltccxmj@sina.com.cn (B.L.); ztyu@hotmail.com (Z.Y.); chen-qi107@163.com (Q.C.)

**Keywords:** elastic properties, anisotropic properties, electronic structure, carbon allotrope, 61.50.-f; 62.20.-x; 63.20.dk; 71.20.-b

## Abstract

$I\bar{4}$–carbon was first proposed by Zhang *et al.*, this paper will report regarding this phase of carbon. The present paper reports the structural and elastic properties of the three-dimensional carbon allotrope $I\bar{4}$–carbon using first-principles density functional theory. The related enthalpy, elastic constants, and phonon spectra confirm that the newly-predicted $I\bar{4}$–carbon is thermodynamically, mechanically, and dynamically stable. The calculated mechanical properties indicate that $I\bar{4}$–carbon has a larger bulk modulus (393 GPa), shear modulus (421 GPa), Young’s modulus (931 GPa), and hardness (55.5 GPa), all of which are all slightly larger than those of c-BN. The present results indicate that $I\bar{4}$–carbon is a superhard material and an indirect-band-gap semiconductor. Moreover, $I\bar{4}$–carbon shows a smaller elastic anisotropy in its linear bulk modulus, shear anisotropic factors, universal anisotropic index, and Young’s modulus.

## 1. Introduction

The group 14 elements, such as carbon, silicon, and germanium, have attracted much interest and have been extensively studied [1,2,3,4,5,6,7,8,9,10,11,12,13,14,15,16,17,18,19,20]. Carbon is found on the Earth mainly in the form of graphite and diamond. The quest for carbon materials with desired properties is of great interest in both fundamental science and advanced technology. One of the most famous carbon materials is graphene. Recently, some scholars of great research found several carbon allotropes with low-energy metastable structures, such as monoclinic M-carbon [1,2], F-carbon [3], orthorhombic W-carbon [4], Z-carbon [5], H-carbon and S-carbon [6], C-carbon [7], Imma-carbon [8,9], M585-carbon [10], T12-carbon [11], C2/m-16 carbon [12], P222_1_-carbon [13], and Cco-carbon [14]. We easily found that these carbon allotropes, with low-energy metastable structures with *sp*^3^ hybridization, could possibly explain the superhard property of materials. Hardness is an important property that determines many of the technological applications of materials. The mechanical properties of some carbon allotropes (such as T-carbon [21] and Y-carbon [22]) are not excellent, as discussed above, but can be improved through modulation [23,24]. At the moment, there are a multitude of metastable carbon allotropes that have been predicted using the quantum-chemical methods of calculation, e.g., evolutionary metadynamics technique [25,26,27,28], and Universal Structure Prediction: Evolutionary Xtallography (USPEX) [29,30,31,32], or post-graphite superhard phase synthesized by cold-compressing graphite [33,34,35,36,37,38,39]. Compressed graphite appears as an initial material for transition to different superhard phases. An ocean of possible carbon modifications can be obtained by combining four-, five-, six-, seven-, even eight-membered carbon rings. The discussion above of carbon allotropes basically adopts this structure. Wang *et al.* [40] found a dynamically stable and energetically favourable carbon allotrope, *i.e.*, J-carbon. It has a wider band gap (5.89 eV within LDA-HSE06) than that of diamond and a larger bulk modulus (395 GPa). Currently, Z-carbon [33,34] is the most stable and hardest material predicted by the theoretical methods compared with other theoretical structures, but it has not exceeded diamond’s bulk modulus and hardness. The inclusion in the Z-carbon of additional diamond blocks generates another superhard carbon allotrope family, investigated in [41]. Li *et al.* [42] predicted a new cubic carbon allotrope, namely, C_96_ carbon. Unfortunately, its mechanical properties are not excellent and it cannot be used as a potential superhard material. Recently, another *sp*^3^ carbon allotrope, *i.e.*, diamond nanothread, was synthesized experimentally; Fitzgibbons *et al.* [43] and Zhan *et al.* [44] then reported regarding its mechanical properties. These nanothreads show extraordinary properties, such as strength and stiffness, higher than those of *sp*^2^ carbon nanotubes. 

Moreover, $I\bar{4}$–carbon was first predicted in [45]. However, the physical properties, *i.e.*, the structural, mechanical, and electronic properties, of $I\bar{4}$–carbon were not studied in [45], nor in other studies. The physical properties of a novel superhard carbon allotrope with tetragonal $I\bar{4}$ symmetry (16 atoms/cell) will be detailed in this paper using first-principles calculations.

## 2. Materials and Methods

The calculations were performed using the density functional theory (DFT) [46,47] by using the Cambridge Serial Total Energy Package (CASTEP) code [48]. The electron–ionic core interaction was represented using the ultrasoft pseudopotentials [49]. The structural optimizations were conducted using the Broyden–Fletcher–Goldfarb–Shanno (BFGS) minimization [50]. The calculations were performed using the local density approximation (LDA) [51,52] and generalized gradient approximation (GGA) in the form of the Perdew–Burke–Ernzerhof (PBE) [53] exchange correlation potential. The total energy convergence tests showed that convergence to within 1 m∙eV/atom was achieved using the above calculation parameters. The electron and core interactions were included by using the ultrasoft pseudopotentials method, with a plane-wave energy cutoff energy of 400 eV, where 2*s*^2^2*p*^2^ are treated as valence electrons for C. Highly dense *k*-point [54] sampling with a grid spacing of less than 2π × 0.025 Å^−1^ (7 × 7 × 7 for $I\bar{4}$–carbon) in the Brillouin zone was used. The self-consistent convergence of the total energy was 5 × 10^−6^ eV/atom; the maximum force on the atom was 0.01 eV/Å; the maximum ionic displacement was within 5 × 10^−4^ Å; the maximum stress was within 0.02 GPa.

## 3. Results and Discussion

### 3.1. Structural Properties

**Figure 1:**
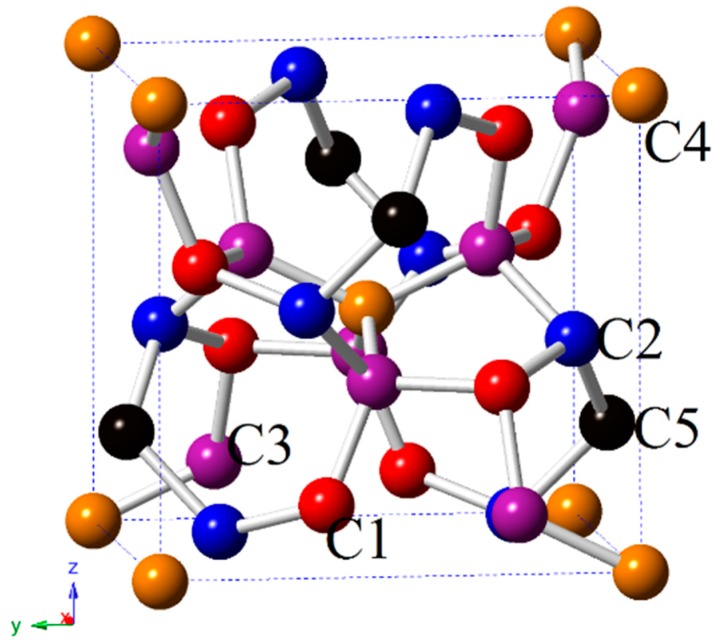
The crystal structure of $I\bar{4}$ –carbon.

There is a new tetragonal carbon phase, namely, $I\bar{4}$–carbon, which belongs to the $I\bar{4}$ space group. The crystal structure of $I\bar{4}$–carbon is shown in Figure 1. Different atomic positions are denoted by different colours of spheres. There are five inequivalent carbon atoms in its conventional cell, located at C1: 8g (−0.1219, −0.2709, −0.6225), C2: 8g (0.4044, −0.1809, 0.0492), C3: 8g (0.4812, 0.2514, −0.3772), C4: 2a (0.5000, 0.5000, 0.50000) and C5: 2d (0.0000, 0.5000, 0.7500). The optimized lattice parameters within the GGA and LDA level of $I\bar{4}$–carbon at ambient pressure are listed in Table 1. As listed, *a* = 5.5628 (5.5017) Å and *c* = 5.5082 (5.4471) Å are within the GGA (LDA) level and c is slightly smaller than a. These lattice parameters are in excellent agreement with the theoretical values predicted by Zhang *et al.* [45]. The results within GGA are closer to the theoretical values predicted by Zhang *et al.* [45]; thus, the following discussions use the results of the GGA level. $I\bar{4}$–carbon has seven bond lengths, which are also listed in Table 1. Between C1 and C2, C1 and C3, there are two different bond lengths, *i.e.*, 1.5586 and 1.5224 Å, 1.5555 and 1.5207 Å, respectively, within the GGA level. The average bond length of $I\bar{4}$–carbon is 1.5510 Å, while it is 1.567 Å and 1.551 Å for C2/m–16 carbon and M carbon, respectively. They are all slightly greater than that of diamond (1.535 Å). The hardness of $I\bar{4}$–carbon, C2/m–16 carbon, M carbon, c-BN and diamond is calculated by using Lyakhov and Oganov’s model [55]. The hardness of $I\bar{4}$–carbon is 55.5 GPa, which is slightly smaller than that of C2/m–16 carbon (59.5 GPa) [12] and M carbon (66.6 GPa), approximately half of that of diamond (89.7 GPa), and slightly larger than that of c-BN (49.9 GPa); the hardness of diamond is very close to the result of Ref [55] (91.0 GPa) using the Lyakhov and Oganov’s model. The hardness of $I\bar{4}$–carbon is 83.0 GPa in Ref [45], the main reason for this situation is that the empirical formula may over or underestimate the value of the material’s hardness. Most researchers agree that “superhard” materials are those with *Hv* exceeding 40 GPa. Although there are slight differences between the results of the empirical models above, all of them greatly exceed 40 GPa, indicating that $I\bar{4}$–carbon is a superhard material.

### 3.2. Mechanical Properties

**Figure 2:**
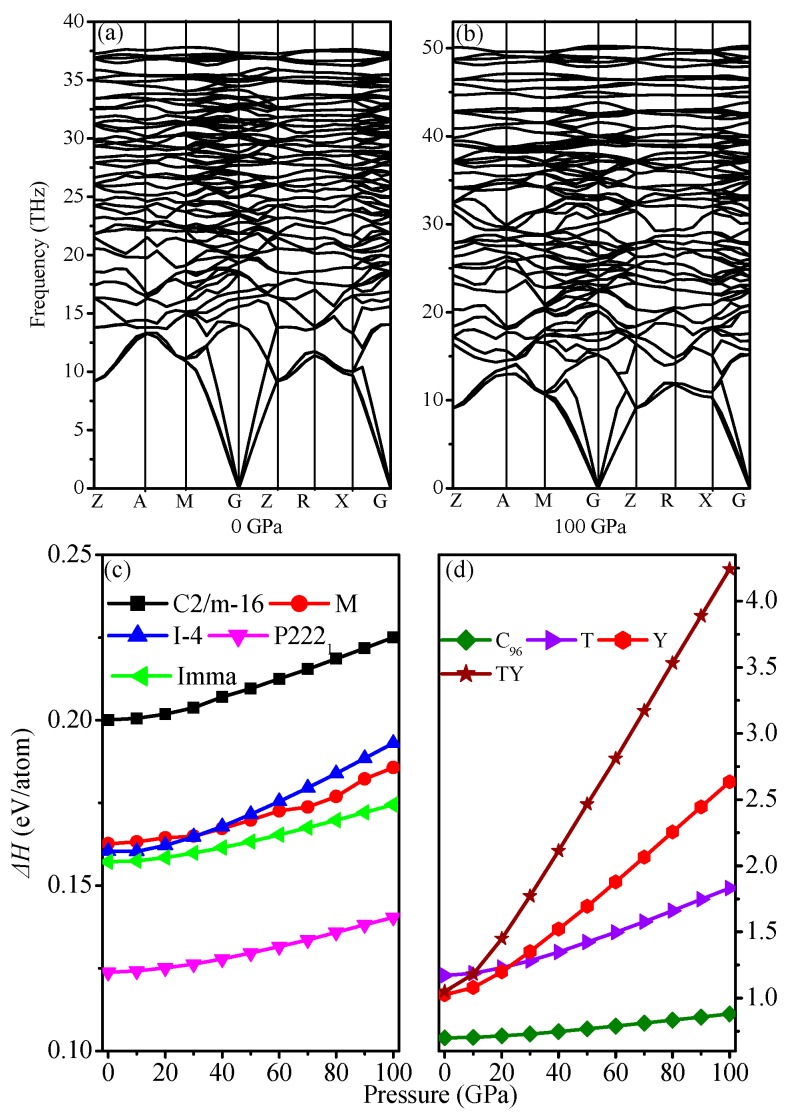
Phonon spectra for $I\bar{4}$ –carbon at 0 GPa (**a**) and 100 GPa (**b**); enthalpies of $I\bar{4}$ –carbon and other carbon allotropes relative to graphite as a function of pressure (**c**,**d**).

For the tetragonal $I\bar{4}$–carbon, seven independent elastic constants *C*_ij_ were determined within the GGA level from the stress of the strained structure with a finite strain. The elastic constants of $I\bar{4}$–carbon under different pressures are listed in Table 2. From Table 2, one can find that the mechanical stability of $I\bar{4}$–carbon satisfies Born’s criterion for a tetragonal crystal [56]: *C*_ii_ > 0, *i* = 1, 3, 4, 6; *C*_11_–*C*_12_ > 0; *C*_11_ + *C*_33_ − 2*C*_13_ > 0 and 2(*C*_11_ + *C*_12_) + *C*_33_ + 4*C*_13_ > 0, indicating that $I\bar{4}$–carbon is mechanically stable at 0–100 GPa. Moreover, almost all elastic constants increase with increasing pressure. *C*_11_ increases the fastest (*dC*_11_/*dP* = 4.57), followed by *C*_33_ (*dC*_33_/*dP* = 4.24); *C*_44_ is the slowest (*dC*44/*dP* = 0.63). *C*_16_ decreases with increasing pressure (*dC*16/*dP* = −0.35). Furthermore, it is important to explore the dynamic stability and thermodynamic stability for further experimental synthesis. The phonon spectra and the enthalpies as a function of pressure are shown in Figure 2a–d. Clearly, no imaginary mode is shown in the phonon spectra. This strongly confirms that the $I\bar{4}$–carbon with the $I\bar{4}$ structure is dynamically stable; thus, it is a new metastable phase in the carbon family. The enthalpy of different carbon allotropes is quantified in terms of the following formation enthalpies formula: *ΔH* = *H*carbon allotropes/*n*_1_ − Hdiamond/*n*_2_, where *n* denotes the number of atoms in a conventional cell for carbon allotropes or graphite. As shown in Figure 2c,d, the calculated formation enthalpies of $I\bar{4}$–carbon are slightly smaller than those of M-carbon below 32.25 GPa. Bulk modulus *B* and shear modulus *G* are calculated by using the Voigt–Reuss–Hill approximation. It is known that the Voigt bound is obtained by using the average polycrystalline modulus, based on an assumption of uniform strain throughout a polycrystalline, and is the upper limit of the actual effective modulus, while the Reuss bound is obtained by assuming a uniform stress and is the lower limit of the actual effective modulus. The arithmetic average of Voigt and Reuss bounds is referred to as the Voigt–Reuss–Hill approximations. Young’s modulus E and Poisson’s ratio v are obtained using the following equations: *E* = 9*BG*/(3*B* + *G*), *v* = (3*B* − 2*G*)/(6*B* + 2*G*), respectively. The calculated results of elastic modulus for $I\bar{4}$–carbon are also shown in Table 2. The bulk modulus, shear modulus, Poisson’s ratio, and Young’s modulus all increase with increasing pressure. The bulk modulus increases the fastest, while shear modulus increases the slowest, *i.e.*, *dB*/*dP* = 3.30 and *dG*/*dP* = 0.86, respectively. Pugh [57] proposed the ratio of bulk to shear modulus (*B*/*G*) and Lewandowski [58] proposed Poisson’s ratio as indications of a ductile *versus* brittle characteristic. *B*/*G* > 1.75 and *v* > 0.26 for a solid material represents ductility, while *B*/*G* < 1.75 and *v* < 0.26 usually indicate brittleness. From 0 to 100 GPa, $I\bar{4}$–carbon is brittle; namely, the brittleness of $I\bar{4}$–carbon decreases with increasing pressure. Compared with other carbon allotropes (P222_1_–carbon: 0.872 [13]; C2/m–16 carbon: 0.867 [12]; Imma–carbon: 0.858 [12]; diamond: 0.831 [12]), $I\bar{4}$–carbon (0.933) has the least brittleness at ambient pressure.

**Figure 3:**
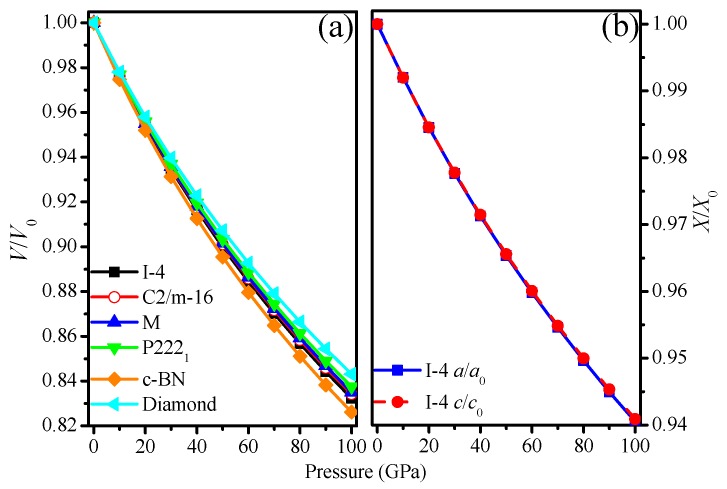
The primitive cell volume *V*/*V*_0_ as a function of pressure for $I\bar{4}$ –carbon, P222_1_-carbon, C2/m-16 carbon, M-carbon, c-BN and diamond (**a**); lattice constants *a*/*a*_0_, *c*/*c*_0_ compression of $I\bar{4}$ –carbon as a function of pressure (**b**).

Incompressibility can reflect the size of the bulk modulus from the other side. The *V*/*V*_0_ of $I\bar{4}$–carbon, C2/m–16 carbon, M–carbon, P222_1_–carbon, c–BN, and diamond as a function of pressure are shown in Figure 3a. From Figure 3a, it is clear that c–BN shows a weaker incompressibility than that of the other carbon allotropes, while $I\bar{4}$–carbon exhibits the weakest incompressibility among the carbon allotropes. The order of incompressibility for carbon allotropes and c-BN is diamond > P222_1_–carbon > M–carbon > C2/m–16 carbon > $I\bar{4}$–carbon > c–BN. The bulk moduli of carbon allotropes and c-BN are also in this order: diamond (439 GPa) > P222_1_–carbon (409 GPa) > M–carbon (399 GPa) > C2/m–16 carbon (398 GPa) > $I\bar{4}$–carbon (393 GPa) > c–BN (370 GPa). Regarding the incompressibility of $I\bar{4}$–carbon in detailed investigations, the lattice parameters *a*/*a*_0_ and c/*c*_0_ as functions of pressure are illustrated in Figure 3b. Regarding the incompressibility of lattice parameters *a*/*a*_0_ and *c*/*c*_0_, they are very close. The incompressibility of lattice parameter *c* is slightly larger than that of *a*. By fitting the calculated data using the polynomial fitting methods, the following relationships at 0 GPa and 0 K are obtained:

*a*/*a*_0_ = 1.96545 × 10^−6^*P*^2^ − 7.85386 × 10^−4^*P +* 0.99970 (1)

*c*/*c*_0_ = 1.93918 × 10^−6^*P*^2^ − 7.79164 × 10^−4^*P +* 0.99965 (2)

where the unit of pressure is GPa. By fitting using the polynomial, it is clear that the incompressibility of lattice parameter *c* is slightly better than that of *a*.

The defined Debye temperature parameter, which can be interpreted as the temperature at which the highest-frequency mode (and hence every mode) is excited, is of great importance, allowing us to predict the heat capacity at any temperature in an atomic solid. The formula for evaluating the Debye temperature is given by [59]:

(3) $$\Theta_{D}=\frac{h}{k_{B}}{\left[\frac{3n}{4\pi }\left(\frac{N_{A}\rho }{M}\right)\right]}^{\frac{1}{3}}v_{m}$$

where *h* denotes Planck’s constant, *k*_B_ denotes Boltzmann’s constant, *n* denotes the number of atoms per formula unit, *N*_A_ denotes Avogadro’s number, *M* denotes molar mass, and *ρ* denotes density. The average sound velocity *v*_m_ is described by the following formula:

(4) $$v_{m}={\left[\frac{1}{3}\left(\frac{2}{v_{t}^{3}}+\frac{1}{v_{l}^{3}}\right)\right]}^{-\frac{1}{3}}$$

**Figure 4:**
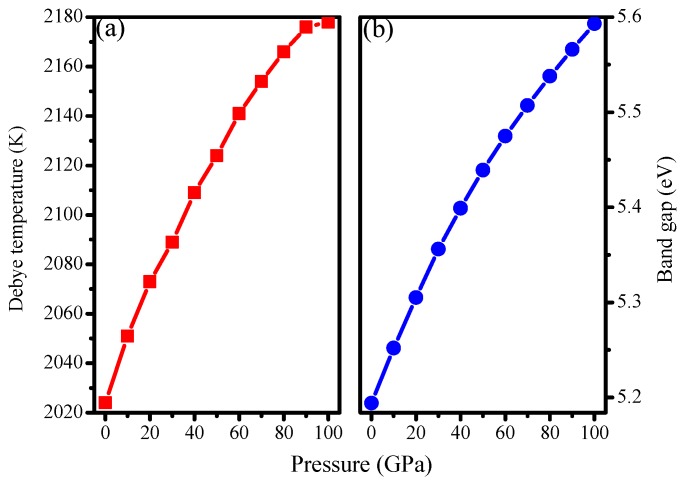
Debye temperature (**a**) and band gap (**b**) of $I\bar{4}$ –carbon as a function of pressure.

The transverse and longitudinal elastic wave velocity, *v*_t_ and *v*_l_, can be obtained using Navier’s equation, as follows [60]:

(5) $$v_{t}=\sqrt{\frac{G}{\rho }}\;\mathrm{and}\;v_{l}=\sqrt{\frac{3B+4G}{3\rho }}$$

where *B* and *G* are the bulk modulus and shear modulus, respectively. Based on Equation (3), the Debye temperature of $I\bar{4}$–carbon *Θ*_D_ = 2024 K. The Debye temperature of $I\bar{4}$–carbon as a function of pressure is plotted in Figure 4a. It is found that the Debye temperature increases with increasing pressure. Furthermore, the greater the pressure, the more slowly the Debye temperature increases. The calculated results of the density, Debye temperature, transverse and longitudinal elastic wave velocity *v*_t_ and *v*_l_, and the mean sound velocity *v*_m_ at different pressures are listed in Table 3. The density and *v*_l_ increase with increasing pressure. With the increase of pressure, the transverse longitudinal elastic wave velocity *v*_t_ shows no monotonic increase or decrease. However, the mean sound velocity *v*_m_ increases with increasing pressure from 0 to 80 GPa and decreases with increasing pressure from 80 to 100 GPa. The main reason is that sometimes *v*_t_ increases with increasing pressure and sometimes decreases, *i.e.*, there is no law.

### 3.3. Anisotropic Properties

It is well known that the anisotropy of elasticity is an important implication in engineering science and crystal physics [61], hence, it is worthwhile to investigate the elastic anisotropy of materials. The shear anisotropic factors provide a measure of *B*_a_, *B*_b_, and *B*_c_, *i.e.*, the bulk modulus along the *a*, *b* and *c* axes, respectively, which can be calculated by using the following equations:

(6) $$B_{a}=a\frac{dP}{da}=\frac{\Lambda }{1+\alpha +\beta },$$

(7) $$B_{b}=b\frac{dP}{db}=\frac{B_{a}}{\alpha },$$

(8) $$B_{c}=c\frac{dP}{dc}=\frac{B_{a}}{\beta },$$

(9) $$\Lambda =C_{11}+2C_{12}\alpha +C_{22}\alpha^{2}+C_{33}\beta^{2}+2C_{13}\beta (1+\alpha ),$$

(10) $$\alpha =\frac{\left(C_{11}-C_{12}\right)}{\left(C_{22}-C_{12}\right)},$$

(11) $$\beta =\frac{\left(C_{22}-C_{12})(C_{11}-C_{13})-(C_{11}-C_{12})(C_{13}-C_{12}\right)}{\left(C_{22}-C_{12})(C_{33}-C_{13}\right)}$$

**Figure 5:**
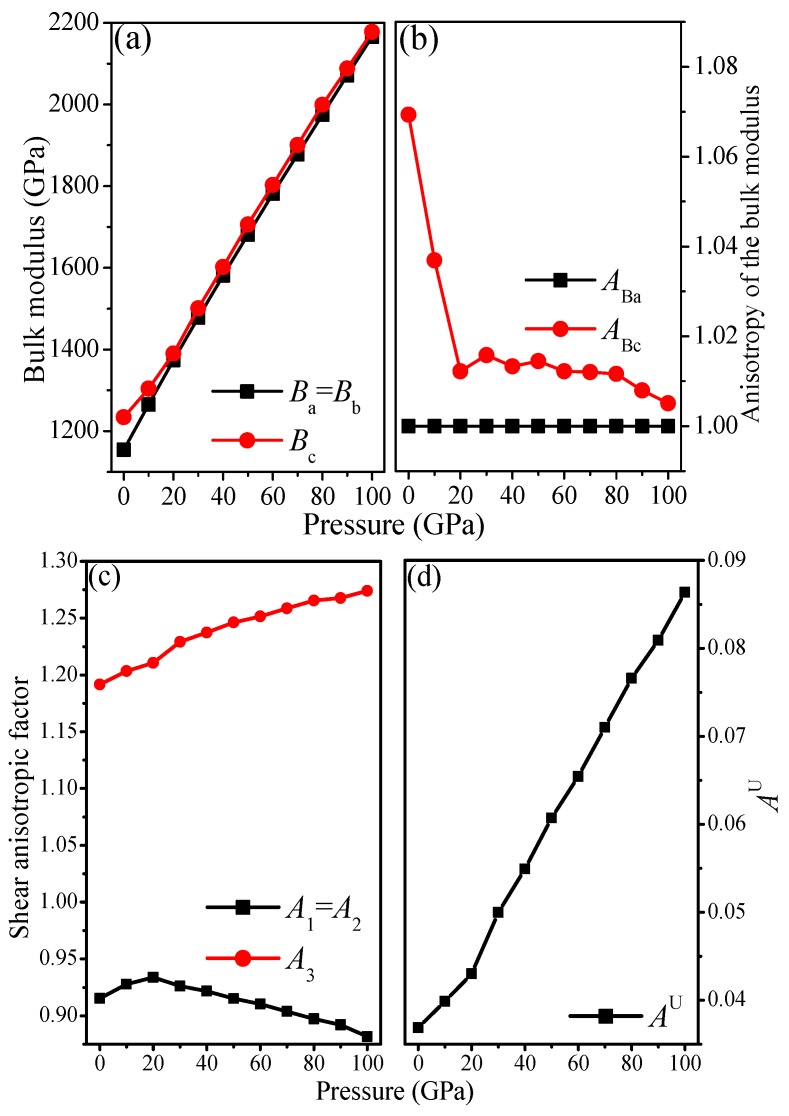
Linear bulk modulus *B*_a_, *B*_b_, and *B*_c_ of $I\bar{4}$ –carbon at 0 K as a function of pressure (**a**); anisotropy factors of $I\bar{4}$ –carbon at 0 K as a function of pressure, (**b**) *A*_Bb_ and *A*_Bc_; (**c**) *A*_1_, *A*_2_ and *A*_3_; (**d**) *A*^U^.

The calculated B_a_, B_b_, and B_c_ at different pressures are shown in Figure 5a. Due to the lattice constant a being equal to b, the bulk modulus along the *a*-axis is equal to that along the *b* axis, in other words, B_a_ = B_b_. It is clear that B_a_, B_b_, and B_c_ increase with increasing pressure and that B_a_, B_b_ (87.69%) increase more than B_c_ (76.50%). The calculated directional bulk modulus suggests that it is the largest along the *a*-axis and the smallest along the *c*-axis, indicating that the compressibility along the *c*-axis is the smallest, while along the *a*-axis, is the largest. This is in accordance with the relationships between the ratios a/a_0_, c/c_0_ and pressure, as shown in Figure 3b. Therefore, the anisotropy of the linear bulk modulus should also be considered. The anisotropy of the bulk modulus along the *a*-axis and *c*-axis with respect to the *b*-axis can be estimated by:

(12) $$A_{B_{a}}=\frac{B_{a}}{B_{b}},\;\mathrm{and}\;A_{B_{c}}=\frac{B_{c}}{B_{b}}$$

The anisotropy factors of the bulk modulus along the *a*-axis and *c*-axis for $I\bar{4}$–carbon at *T* = 0 K as a function of pressure are shown in Figure 5b. Note that a value of 1.0 indicates elastic isotropy and any departure from 1.0 represents elastic anisotropy. The anisotropy of the bulk modulus along the *a*-axis and *c*-axis with respect to the *b*-axis shows that *A*_Ba_ is elastic isotropy and *A*_Bc_ is elastic anisotropy. 

After discussing the anisotropy of the bulk modulus, we now discuss the anisotropy of the shear modulus. The shear anisotropic factors provide a measure of the degree of anisotropy in the bonding between atoms in different planes. The shear anisotropic factor for the (1 0 0) shear planes between [0 1 1] and [0 1 0] directions and the (0 1 0) shear planes between [1 0 1] and [0 0 1] directions is [62]:

(13) $$A_{1}=\frac{4C_{44}}{C_{11}+C_{33}-2C_{13}},$$

For the (0 1 0) shear planes between [1 0 1] and [0 0 1] directions, it is [60]:

(14) $$A_{2}=\frac{4C_{55}}{C_{22}+C_{33}-2C_{23}},$$

For the (0 0 1) shear planes between [1 1 0] and [0 1 0] directions, it is [60]:

(15) $$A_{3}=\frac{4C_{66}}{C_{11}+C_{22}-2C_{12}}$$

For an isotropic crystal, the factors *A*_1_, *A*_2_, and *A*_3_ are 1.0, while any value smaller or larger than 1.0 is a measure of the elastic anisotropy possessed by the materials. The anisotropy factors of $I\bar{4}$–carbon at *T* = 0 K as a function of pressure are shown in Figure 5c. Due to the symmetry of the crystal structure, *C*_11_ = *C*_22_, *C*_44_ = *C*_55_ and *C*_13_ = *C*_23_; thus, *A*_1_ = *A*_2_. From Figure 5c, A_1_ first increases and then decreases with increasing pressure, while *A*_3_ increases monotonically with increasing pressure. $I\bar{4}$–carbon shows elastic anisotropy in the bulk modulus and shear modulus. The universal anisotropic index (*A*^U^ = 5G_V_/*G*_R_ + *B*_V_/*B*_R_ − 6) combines the shear modulus and bulk modulus to exhibit the anisotropy of the material. The *A*^U^ of $I\bar{4}$–carbon increases with pressure (see Figure 5d), indicating that the anisotropy of $I\bar{4}$–carbon increases with increasing pressure. The *A*^U^ of $I\bar{4}$–carbon is 0.0369, which is smaller than that of C2/m–carbon (0.0766) and P222_1_–carbon (0.0526).

**Figure 6:**
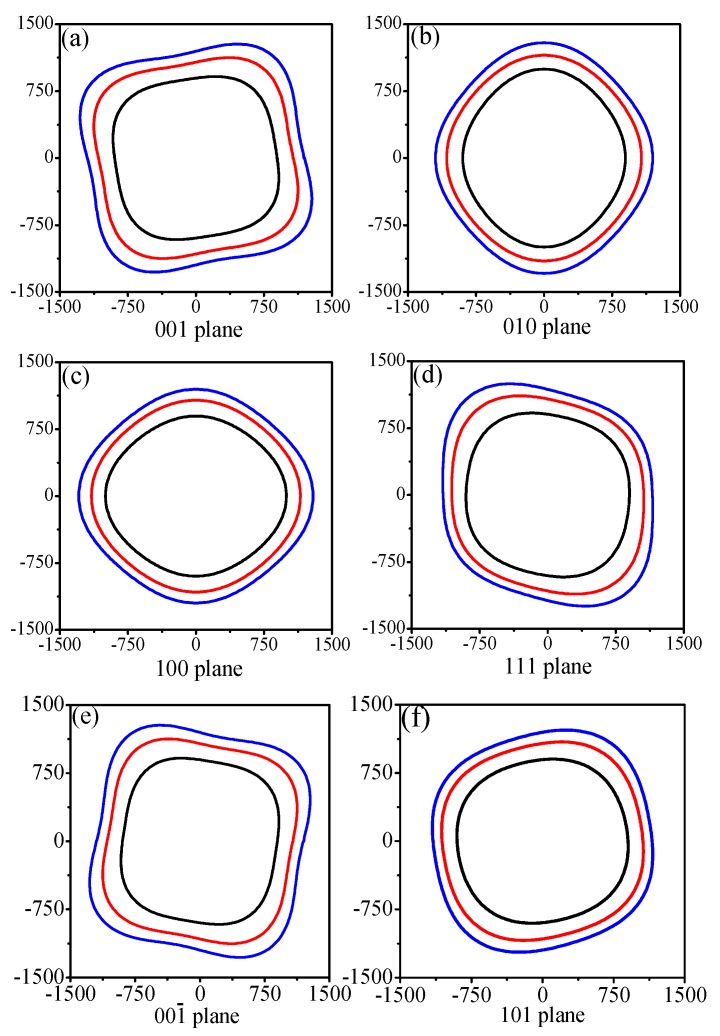
2D representation of Young’s modulus in the (001), (010), (100), (111), ($00\bar{1}$ ), and (101) planes for $I\bar{4}$ –carbon, shown in (**a**–**f**), respectively.

Young’s modulus is not always the same in all orientations of a material; it will change depending on the direction of the force vector. Engineers can use this directional phenomenon to their advantage in creating structures. The directional dependence of the anisotropy in Young’s modulus is calculated by using the Elastic Anisotropy Measures (ELAM) [63,64] code. The 2D representation of Young’s modulus for $I\bar{4}$–carbon in the (001), (010), (100), (111), ($00\bar{1}$), and (101) planes is shown in Figure 6a–f, respectively. The black, red, and blue solid curves represent Young’s modulus for $I\bar{4}$–carbon in the (001), (010), (100), (111), ($00\bar{1}$), and (101) planes at 0, 50 and 100 GPa, respectively. It is obvious that the anisotropy of Young’s modulus increases with increasing pressure in the (001), (010), (100), (111), ($00\bar{1}$), and (101) planes. To investigate the anisotropy of Young’s modulus in detail, we calculate the maximal and minimal values of Young’s modulus for $I\bar{4}$–carbon in the (001), (010), (100), (111), ($00\bar{1}$), and (101) planes and for all possible directions, together with the ratio *E*_max_/*E*_min_, which are listed in Table 4. The conclusion from Table 4 is consistent with the conclusion we obtained from Figure 6, *i.e.*, that the anisotropy of Young’s modulus increases with increasing pressure in the (001), (010), (100), (111), ($00\bar{1}$), and (101) planes. Regarding the distribution of Young's modulus in the (001) plane and ($00\bar{1}$) plane, the maximal and minimal values are the same, while in the (010) plane and (100) plane, they are also the same. The (001) plane and ($00\bar{1}$) plane exhibit the largest anisotropy of Young’s modulus for $I\bar{4}$–carbon, while the (101) plane exhibits the smallest anisotropy of Young’s modulus. This situation also appears under high pressure. All of the degrees of special plane anisotropy are smaller than the overall performance of the material anisotropy because the minimal value of the entire Young’s modulus was not obtained. The anisotropy of Young’s modulus of $I\bar{4}$–carbon (*E*_max_/*E*_min_ = 1.133) is slightly smaller than that of C2/m-16 carbon (*E*_max_/*E*_min_ = 1.275) and P2221-carbon (*E*_max_/*E*_min_ = 1.173). Thus, $I\bar{4}$–carbon shows a smaller elastic anisotropy in its linear bulk modulus, shear anisotropic factors, universal anisotropic index, and Young’s modulus.

### 3.4. Electronic Properties

**Figure 7:**
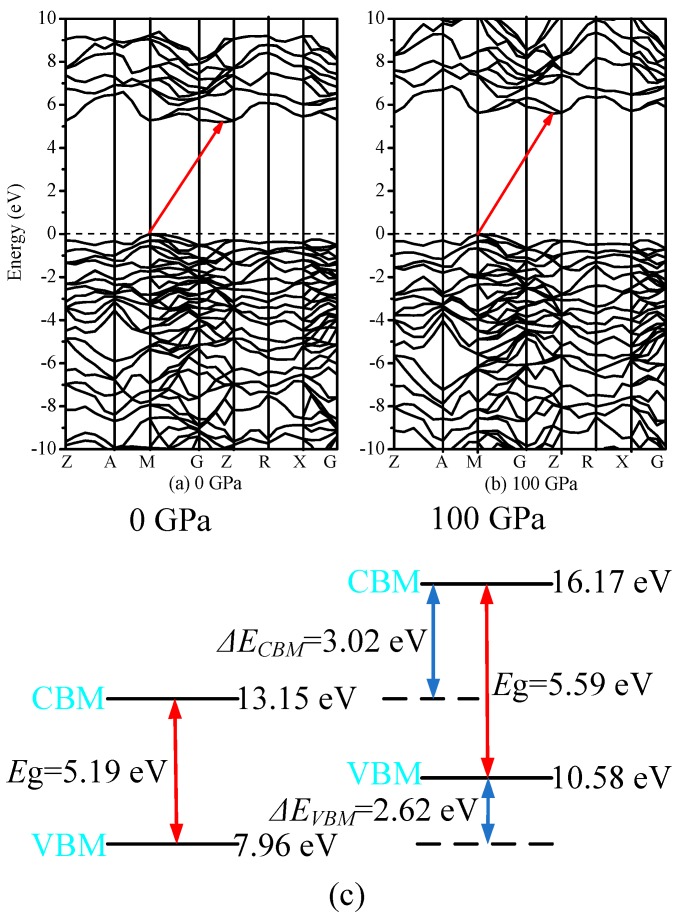
Electronic band structure for the $I\bar{4}$ –carbon at ambient pressure (**a**) and 100 GPa**** (**b**); the calculated energy values of CBM and VBM under different pressures (**c**).

**Figure 8:**
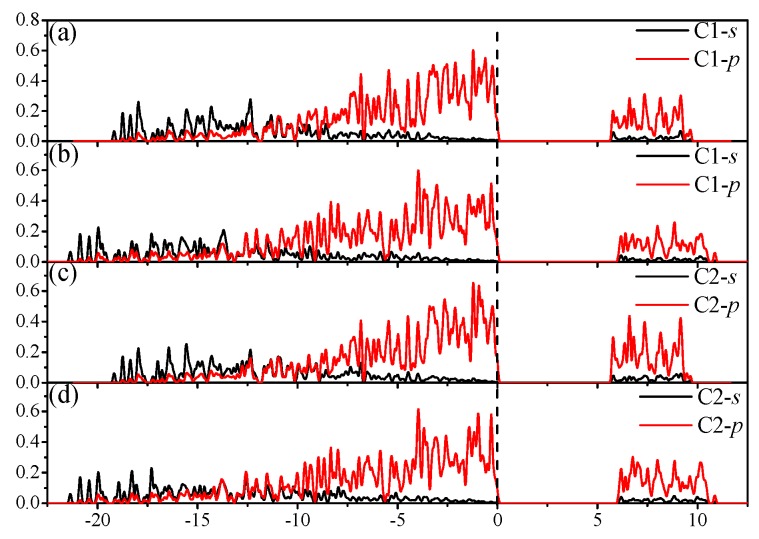

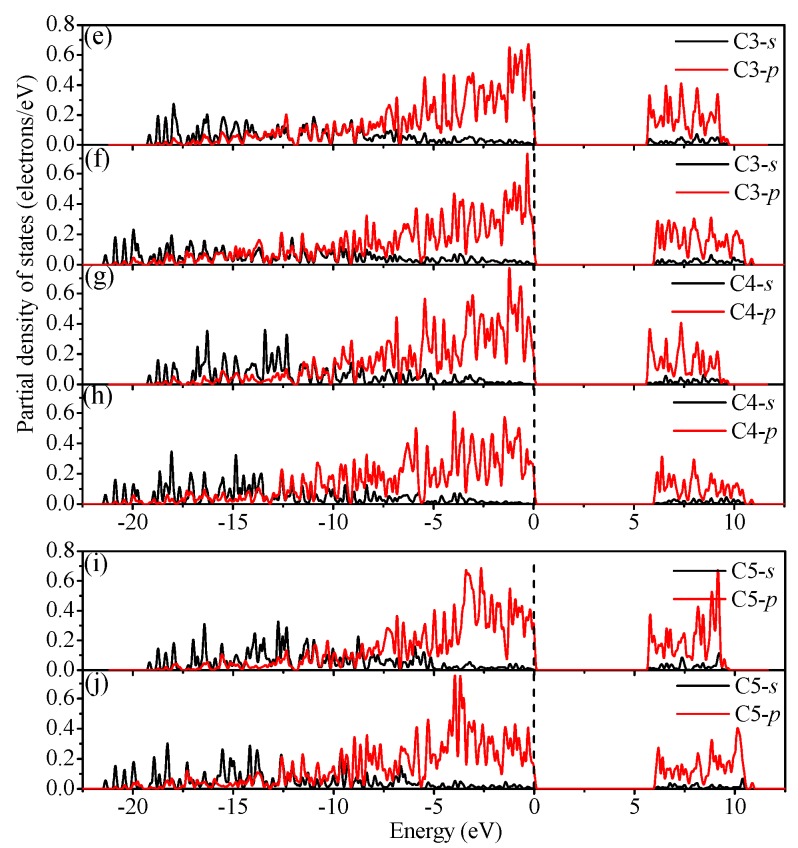
Partial density of state for $I\bar{4}$ –carbon; (**a**,**c**,**e**,**g**,**i**) are at ambient pressure, while (**b**,**d**,**f**,**h**,**j**) are at 100 GPa.

The band structures of $I\bar{4}$–carbon are shown in Figure 7a,b at 0 and 100 GPa, respectively. Semiconductor materials have direct or indirect band gaps. In direct band gap materials, the maximum of the valence band (VBM) and minimum of the conduction band (CBM) occur at the same value of the wave vector, while in indirect band gap materials, they occur for different values of the wave vector. It is obvious that $I\bar{4}$–carbon is an indirect-band-gap and wide-band-gap semiconductor material. The VBM of $I\bar{4}$–carbon is located at the M point (0.500, 0.500, 0.000), while the CBM is located from point (0.000, 0.000, 0.3667) through G (0.000, 0.000, 0.000) to the Z point (0.000, 0.000, 0.500), whether at ambient pressure or under high pressure. From Figure 7a,b, it is shown that the band gap increases with increasing pressure; more details are shown in Figure 4b; the band gap of $I\bar{4}$–carbon increases from 5.19 eV to 5.59 eV. The greater the pressure, the less the band gap increases. To determine the reason why, we calculate the energy values of the CBM and VBM under different pressures. The calculated results are illustrated in Figure 7c. The energy of VBM for $I\bar{4}$–carbon is 7.96 eV at 0 GPa and 10.58 eV at 100 GPa; *ΔE*_VBM_ = 2.62 eV. The energy of CBM for $I\bar{4}$–carbon is 13.15 eV at 0 GPa and 16.17 eV at 100 GPa; *ΔE*_CBM_ = 3.02 eV. *ΔE*_CBM_ − *ΔE*_VBM_ = 0.40 eV, which is the same as with the band gap increase. In other words, as pressure increases, the conduction band and valence band energy will increase, but the energy of the conduction band will increase more than that of the valence band. The partial density of states (PDOS) of five inequivalent carbon atoms in $I\bar{4}$–carbon at 0 and 100 GPa are shown in Figure 8. Figure 8a,c,e,g,i show the partial density of states of five inequivalent carbon atoms at 0 GPa; Figure 8b,d,f,h,j show the partial density of states of five inequivalent carbon atoms at 100 GPa. No matter the position of the carbon atoms, the partial density of states in the conduction band part is, with increasing pressure intensity, near low energy, and the valence band deviates from the Fermi level. The PDOS in the lower energy part is contributed mainly by the C-2*s* orbitals, while in the other energy part, is contributed mainly by the C-2*p* orbitals.

## 4. Conclusions

In conclusion, the low-energy metastable structures of $I\bar{4}$–carbon have been systematically investigated based on the density functional theory. The related enthalpy, elastic constants and phonon spectra confirm that the newly-predicted $I\bar{4}$–carbon is thermodynamically, mechanically, and dynamically stable. As a potential superhard material for engineering applications, the mechanical properties of $I\bar{4}$–carbon indicate that it is a superhard material. $I\bar{4}$–carbon shows a smaller anisotropy in its linear bulk modulus, shear anisotropic factors, universal anisotropic index, and Young’s modulus. The band structure shows that $I\bar{4}$–carbon is an indirect-band-gap and wide-band-gap semiconductor material. Finally, the calculations of its mechanical properties reveal that $I\bar{4}$–carbon possesses a high bulk and shear modulus as well as a low Poisson’s ratio and *B*/*G* ratio (<1.75). Moreover, $I\bar{4}$–carbon has a larger Debye temperature (*Θ*_D_ = 2024 K). Due to their wide-band-gap and higher bulk moduli, hardness, they are attractive for semiconductor device applications and superhard material with potential technological and industrial applications.

## Figures and Tables

**Table 1:** The calculated lattice parameters (in Å) and bond lengths (in Å) for $I\bar{4}$ –carbon.

Methods	*a*	*c*	*d*_C1-C2_	*d*_C1-C3_	*d*_C2-C3_	*d*_C2-C5_	*d*_C3-C4_
GGA	5.5628	5.5082	1.5586	1.5555	1.5694	1.5871	1.5430
			1.5224	1.5207			
LDA	5.5017	5.4471	1.5435	1.5408	1.5531	1.5683	1.5253
			1.5036	1.5039			

**Table 2:** The calculated elastic constants (in GPa) and elastic modulus (in GPa) of $I\bar{4}$ –carbon at different pressures.

P	*C*_11_	*C*_12_	*C*_13_	*C*_16_	*C*_33_	*C*_44_	*C*_66_	*B*	*G*
0	926	133	101	−28	1017	398	472	393	421
10	1015	175	142	−35	1028	408	493	441	434
20	1034	189	153	−40	1081	422	511	460	446
30	1079	220	182	−43	1132	428	528	495	455
40	1128	248	208	−46	1181	436	544	529	466
50	1174	274	235	−49	1229	442	559	563	475
60	1220	304	261	−53	1275	449	573	596	484
70	1263	332	287	−56	1320	454	586	629	491
80	1306	360	303	−59	1364	458	598	661	499
90	1347	387	340	−62	1403	462	608	692	504
100	1383	419	366	−63	1441	461	614	723	507

**Table 3:** The calculated density (*ρ*, in g/cm^3^), the longitudinal, transverse, and mean elastic wave velocity (*v*_s_, *v*_p_, *v*_m_, in m/s), and the Debye temperature (*Θ*_D_, in K) for $I\bar{4}$ –carbon.

P	*ρ*	*v_l_*	*v_t_*	*v_m_*	*Θ*_D_
0	3.2764	17,069	11,336	12,398	2024
10	3.3564	17,434	11,373	12,467	2051
20	3.4332	17,529	11,402	12,503	2073
30	3.5059	17,730	11,395	12,513	2089
40	3.5750	17,939	11,415	12,549	2109
50	3.6412	18124	11417	12566	2124
60	3.7045	18303	11427	12589	2141
70	3.7654	18465	11424	12598	2154
80	3.8243	18617	11417	12602	2166
90	3.8814	18751	11400	12594	2176
100	3.9365	18852	11345	12547	2178

**Table 4:** The maximum and minimum values of Young’s modulus (in GPa) in different planes and for pressures for $I\bar{4}$ –carbon.

Plane	*P*	Maximum	Minimum	*E*_max_/*E*_min_
(001)	0	999	887	1.126
	50	1250	1054	1.186
	100	1427	1168	1.222
(010)	0	998	889	1.123
	50	1153	1053	1.095
	100	1292	1150	1.123
(100)	0	998	889	1.123
	50	1153	1053	1.095
	100	1292	1150	1.123
(111)	0	992	888	1.117
	50	1233	1045	1.180
	100	1402	1139	1.231
($00\bar{1}$)	0	999	887	1.126
	50	1250	1054	1.186
	100	1427	1168	1.222
(101)	0	949	885	1.072
	50	1160	1050	1.105
	100	1298	1147	1.132
All	0	999	882	1.133
	50	1250	1042	1.200
	100	1427	1138	1.254

## References

[1] Oganov A.R., Glass C.W. (2006). Crystal structure prediction using ab initio evolutionary techniques: Principles and applications. J. Chem. Phys..

[2] Li Q., Ma Y.M., Oganov A.R., Wang H.B., Wang H., Xu Y., Cui T., Mao H.K., Zou G.T. (2009). Superhard Monoclinic Polymorph of Carbon. Phys. Rev. Lett..

[3] Tian F., Dong X., Zhao Z.S., He J.L., Wang H.T. (2012). Superhard F-carbon predicted by ab initio particle-swarm optimization methodology. J. Phys. Condens. Matter.

[4] Wang J.T., Chen C., Kawazoe Y. (2011). Low-Temperature Phase Transformation from Graphite to *sp*^3^ Orthorhombic Carbon. Phys. Rev. Lett..

[5] Li Z.P., Gao F.M., Xu Z.M. (2012). Strength, hardness, and lattice vibrations of Z-carbon and W-carbon: First-principles calculations. Phys. Rev. B.

[6] He C.Y., Sun L.Z., Zhang C.X., Peng X.Y., Zhang K.W., Zhong J.X. (2012). New superhard carbon phases between graphite and diamond. Solid State Commun..

[7] Li D., Bao K., Tian F.B., Zeng Z.W., He Z., Liu B.B., Cui T. (2012). Lowest enthalpy polymorph of cold-compressed graphite phase. Phys. Chem. Chem. Phys..

[8] Wei Q., Zhang M.G., Yan H.Y., Lin Z.Z., Zhu X.M. (2014). Structural, electronic and mechanical properties of Imma-carbon. EPL.

[9] Liu Y.M., Lu M.C., Zhang M. (2014). First-principles study of a novel superhard *sp*^3^ carbon allotrope. Phys. Lett. A.

[10] He C.Y., Zhong J.X. (2014). M585, a low energy superhard monoclinic carbon phase. Solid State Commun..

[11] Zhao Z.S., Tian F., Dong X., Li Q., Wang Q.Q., Wang H., Zhong X., Xu B., Yu D.L., He J.L. (2012). Tetragonal Allotrope of Group 14 Elements. J. Am. Chem. Soc..

[12] Xing M.J., Li B.H., Yu Z.T., Chen Q. (2015). C2/m-carbon: Structural, mechanical, and electronic properties. J. Mater. Sci..

[13] Xing M.J., Li B.H., Yu Z.T., Chen Q. (2015). Structural, Elastic, and Electronic Properties of a New Phase of Carbon. Commun. Theor. Phys..

[14] Zhao Z.S., Xu B., Zhou X.F., Wang L.M., Wen B., He J.L., Liu Z.Y., Wang H.T., Tian Y.J. (2011). Novel Superhard Carbon: C-Centered Orthorhombic C_8_. Phys. Rev. Lett..

[15] Guo Y.G., Wang Q., Kawazoe Y., Jena P. (2015). A New Silicon Phase with Direct Band Gap and Novel Optoelectronic Properties. Sci. Rep..

[16] Fan Q.Y., Chai C.C., Wei Q., Yan H.Y., Zhao Y.B., Yang Y.T., Yu X.H., Liu Y., Xing M.J., Zhang J.Q. (2015). Novel silicon allotropes: Stability, mechanical, and electronic properties. J. Appl. Phys..

[17] Lee I.H., Lee J.Y., Oh Y.J., Kim S., Chang K.J. (2014). Computational search for direct band gap silicon crystals. Phys. Rev. B.

[18] Fan Q.Y., Chai C.C., Wei Q., Yang Y.T., Yang Q., Chen P.Y., Xing M.J., Zhang J.Q., Yao R.H. (2016). Prediction of novel phase of silicon and Si–Ge alloys. J. Solid State Chem..

[19] De A., Pryor C.E. (2014). Electronic structure and optical properties of Si, Ge and diamond in the lonsdaleite phase. J. Phys. Condens. Matter.

[20] Wang Q.Q., Xu B., Sun J., Liu H.Y., Zhao Z.S., Yu D.L., Fan C.Z., He J.L. (2014). Direct Band Gap Silicon Allotropes. J. Am. Chem. Soc..

[21] Sheng X.L., Yan Q.B., Ye F., Zheng Q.R., Su G. (2011). T-Carbon: A Novel Carbon Allotrope. Phys. Rev. Lett..

[22] Jo J.Y., Kim B.G. (2012). Carbon allotropes with triple bond predicted by first-principle calculation: Triple bond modified diamond and T-carbon. Phys. Rev. B.

[23] Srinivasu K., Ghosh S.K. (2012). Electronic Structure, Optical Properties, and Hydrogen Adsorption Characteristics of Supercubane-Based Three-Dimensional Porous Carbon. J. Phys. Chem. C.

[24] Li D., Tian F.B., Duan D.F., Zhao Z.L., Liu Y.X., Chu B.H., Sha X.J., Wang L., Liu B.B., Cui T. (2014). Modulated T carbon-like carbon allotropes: An ab initio study. RSC Adv..

[25] Martoňák R., Oganov A.R., Glass C.W. (2007). Crystal structure prediction and simulations of structural transformations: Metadynamics and evolutionary algorithms. Phase Transit..

[26] Zhu Q., Oganov A.R., Lyakhov A.O. (2012). Evolutionary metadynamics: A novel method to predict crystal structures. Crystengcomm.

[27] Boulfelfel S.E., Zhu Q., Oganov A.R. (2012). Novel *sp*^3^ forms of carbon predicted by evolutionary metadynamics and analysis of their synthesizability using transition path sampling. J. Superhard Mater..

[28] Finkelstein G.J., Dera P.K., Jahn S., Oganov A.R., Holl C.M., Meng Y., Duffy T.S. (2014). Phase transitions and equation of state of forsterite to 90 GPa from single-crystal X-ray diffraction and molecular modelling. Am. Mineral..

[29] Oganov A.R., Glass C.W. (2008). Evolutionary crystal structure prediction as a tool in materials design. J. Phys. Condens. Matter.

[30] Oganov A.R., Ma Y.M., Lyakhov A.O., Valle M., Gatti C. (2010). Evolutionary Crystal Structure Prediction as a Method for the Discovery of Minerals and Materials. Theor. Comput. Methods Miner. Phys. Geophys. Appl. Rev. Miner. Geochem..

[31] Oganov A.R., Lyakhov A.O., Valle M. (2011). How Evolutionary Crystal Structure Prediction Works—And Why. Accounts Chem. Res..

[32] Lyakhov A.O., Oganov A.R., Stokes H.T., Zhu Q. (2013). New developments in evolutionary structure prediction algorithm USPEX. Comput. Phys. Commun..

[33] Selli D., Baburin I.A., Martonák R., Leoni S. (2011). Superhard *sp*^3^ carbon allotropes with odd and even ring topologies. Phys. Rev. B.

[34] Amsler M., Flores-Livas J.A., Lehtovaara L., Balima F., Ghasemi S.A., Machon D., Pailhès S., Willand A., Caliste D., Botti S. (2012). Crystal Structure of Cold Compressed Graphite. Phys. Rev. Lett..

[35] Wang J.T., Chen C., Kawazoe Y. (2012). Orthorhombic carbon allotrope of compressed graphite: *Ab initio* calculations. Phys. Rev. B.

[36] He C.Y., Sun L.Z., Zhang C.X., Zhong J.X. (2013). Two viable three-dimensional carbon semiconductors with an entirely *sp*^2^ configuration. Phys. Chem. Chem. Phys..

[37] He C.Y., Sun L.Z., Zhang C.X., Peng X.Y., Zhang K.W., Zhong J.X. (2012). Four superhard carbon allotropes: A first-principles study. Phys. Chem. Chem. Phys..

[38] Kvashnina Y.A., Kvashnin A.G., Sorokin P.B. (2013). Investigation of new superhard carbon allotropes with promising electronic properties. J. Appl. Phys..

[39] Xie H.X., Yin F.X., Yu T. (2014). Mechanism for direct graphite-to-diamond phase transition. Sci. Rep..

[40] Wang J.T., Chen C.F., Kawazoe Y. (2012). Phase conversion from graphite toward a simple monoclinic *sp*^3^-carbon allotrope. J. Chem. Phys..

[41] Zhou R., Zeng X.C. (2012). Polymorphic Phases of *sp*^3^-hybridized carbon under cold compression. J. Am. Chem. Soc..

[42] Li D., Tian F.B., Chu B.H., Duan D.F., Wei S.L., Lv Y.Z., Zhang H.D., Wang L., Lu N., Liu B.B. (2015). Cubic C_96_: A novel carbon allotrope with a porous nanocube network. J. Mater. Chem. A.

[43] Fitzgibbons T.C., Guthrie M., Xu E.S., Crespi V.H., Davidowski S.K., Cody G.D., Alem N., Badding J.V. (2015). Benzene-derived carbon nanothreads. Nat. Mater..

[44] Zhan H.F., Zhang G., Tan V.B.C., Cheng Y., Bell J.M., Zhang Y.W., Gu Y.T. (2016). From Brittle to Ductile: A Structure Dependent Ductility of Diamond Nanothread. Nanoscale.

[45] Zhang X.X., Wang Y.C., Lv J., Zhu C.Y., Li Q., Zhang M., Li Q., Ma Y.M. (2013). First-principles structural design of superhard materials. J. Chem. Phys..

[46] Hohenberg P., Kohn W. (1964). Inhomogeneous electron gas. Phys. Rev..

[47] Kohn W., Sham L.J. (1965). Self-consistent equations including exchange and correlation effects. Phys. Rev..

[48] Clark S.J., Segall M.D., Pickard C.J., Hasnip P.J., Probert M.I.J., Refson K., Payne M.C. (2005). First principles methods using CASTEP. Z. Kristallogr..

[49] Vanderbilt D. (1990). Soft self-consistent pseudopotentials in a generalized eigenvalue formalism. Phys. Rev. B.

[50] Pfrommer B.G., Côté M., Louie S.G., Cohen M.L. (1997). Relaxation of crystals with the quasi-newton method. J. Comput. Phys..

[51] Ceperley D.M., Alder B.J. (1980). Ground state of the electron gas by a stochastic method. Phys. Rev. Lett..

[52] Perdew J.P., Zunger A. (1981). Self-interaction correction to density-functional approximations for many-electron systems. Phys. Rev. B.

[53] Perdew J.P., Burke K., Ernzerhof M. (1996). Generalized gradient approximation made simple. Phys. Rev. Lett..

[54] Monkhorst H.J., Pack J.D. (1976). Special points for Brillouin-zone integrations. Phys. Rev. B.

[55] Lyakhov A.O., Oganov A.R. (2011). Evolutionary search for superhard materials: Methodology and applications to forms of carbon and TiO_2_. Phys. Rev. B.

[56] Wu Z.J., Zhao E.J., Xiang H.P., Hao X.F., Liu X.J., Meng J. (2007). Crystal structures and elastic properties of superhard IrN_2_ and IrN_3_ from first principles. Phys. Rev. B.

[57] Pugh S.F. (1954). XCII. Relations between the elastic moduli and the plastic properties of polycrystalline pure metals. Lond. Edinb. Dublin Philos. Mag. J. Sci. Ser. 7.

[58] Lewandowski J.J., Wang W.H., Greer A.L. (2005). Intrinsic plasticity or brittleness of metallic glasses. Philos. Mag. Lett..

[59] Anderson O.L. (1963). A simplified method for calculating the debye temperature from elastic constants. J. Phys. Chem. Solids.

[60] Panda K.B., Ravi K.S. (2006). Determination of elastic constants of titanium diboride (TiB_2_) from first principles using FLAPW implementation of the density functional theory. Comput. Mater. Sci..

[61] Fan Q.Y., Wei Q., Yan H.Y., Zhang M.G., Zhang Z.X., Zhang J.Q., Zhang D.Y. (2014). Elastic and electronic properties of Pbca-BN: First-principles calculations. Comput. Mater. Sci..

[62] Connetable D., Thomas O. (2009). First-principles study of the structural, electronic, vibrational, and elastic properties of orthorhombic NiSi. Phys. Rev. B.

[63] Marmier A., Lethbridge Z.A.D., Walton R.I., Smith C.W., Parker S.C., Evans K.E. (2010). ElAM: A computer program for the analysis and representation of anisotropic elastic properties. Comput. Phys. Commun..

[64] Fan Q.Y., Wei Q., Chai C.C., Yan H.Y., Zhang M.G., Lin Z.Z., Zhang Z.X., Zhang J.Q., Zhang D.Y. (2015). Structural, mechanical, and electronic properties of P3m1-BCN. J. Phys. Chem. Solids.

